# Prevention and Control of Pathogens Based on Big-Data Mining and Visualization Analysis

**DOI:** 10.3389/fmolb.2020.626595

**Published:** 2021-02-25

**Authors:** Cui‐Xia Chen, Li‐Na Sun, Xue‐Xin Hou, Peng‐Cheng Du, Xiao‐Long Wang, Xiao‐Chen Du, Yu‐Fei Yu, Rui‐Kun Cai, Lei Yu, Tian‐Jun Li, Min‐Na Luo, Yue Shen, Chao Lu, Qian Li, Chuan Zhang, Hua‐Fang Gao, Xu Ma, Hao Lin, Zong‐Fu Cao

**Affiliations:** ^1^ National Research Institute for Family Planning, Beijing, China; ^2^ National Center of Human Genetic Resources, Beijing, China; ^3^ National Institute for Communicable Disease Control and Prevention, Beijing, China; ^4^ Bejing Ditan Hospital, Beijing, China; ^5^ Aerospace Information Research Institute, Chinese Academy of Sciences, Beijing, China; ^6^ Shanghai Jiaotong University School of Medicine, Shanghai, China; ^7^ Center for Informational Biology, University of Electronic Science and Technology of China, Chengdu, China

**Keywords:** big data mining, visualization, pathogen identification, genome analysis, virulence, drug-resistance

## Abstract

Morbidity and mortality caused by infectious diseases rank first among all human illnesses. Many pathogenic mechanisms remain unclear, while misuse of antibiotics has led to the emergence of drug-resistant strains. Infectious diseases spread rapidly and pathogens mutate quickly, posing new threats to human health. However, with the increasing use of high-throughput screening of pathogen genomes, research based on big data mining and visualization analysis has gradually become a hot topic for studies of infectious disease prevention and control. In this paper, the framework was performed on four infectious pathogens (Fusobacterium, Streptococcus, Neisseria, and Streptococcus salivarius) through five functions: 1) genome annotation, 2) phylogeny analysis based on core genome, 3) analysis of structure differences between genomes, 4) prediction of virulence genes/factors with their pathogenic mechanisms, and 5) prediction of resistance genes/factors with their signaling pathways. The experiments were carried out from three angles: phylogeny (macro perspective), structure differences of genomes (micro perspective), and virulence and drug-resistance characteristics (prediction perspective). Therefore, the framework can not only provide evidence to support the rapid identification of new or unknown pathogens and thus plays a role in the prevention and control of infectious diseases, but also help to recommend the most appropriate strains for clinical and scientific research. This paper presented a new genome information visualization analysis process framework based on big data mining technology with the accommodation of the depth and breadth of pathogens in molecular level research.

## Introduction

Data published by the World Health Organization shows that the incidence and mortality of human infectious diseases rank highest among all human illness (Gilmour et al., 2013). Emerging and reappearing pathogenic infections occur constantly, with many pathogenic mechanisms remaining unclear (Karesh et al., 2012). Misuse of a large number of broad-spectrum antibiotics has caused strong selection pressure leading to the mutation and rapid variation of pathogens. As a result, the emergence of drug-resistant bacterial strains poses new threats to human health, while the rapid mutation of pathogens is a huge obstacle for design and long term efficacy of vaccines. For example, the emergence of drug-resistant Tuberculosis bacilli (TB) has allowed previously controlled TB infections to become rampant around the world (Cole, 2002).

With the maturation of sequencing technology, large-scale sequencing and even whole-genome sequencing of pathogens has become an important method for research, prevention and control of infectious pathogens. Therefore, big data mining based on genomes has become a hot issue in computational biology. It aims to research ways to explore the phylogenetic laws of infectious pathogens, to monitor changes in pathogen genomes timeously, and to quickly and effectively identify pathogens new or unknown. Information visualization has increasingly become an important research direction but remains a challenge for the prevention and control of infectious diseases (Mao Ping and Wang, 2017; Kan et al., 2018; Chen et al., 2020b).

**FIGURE 1:**
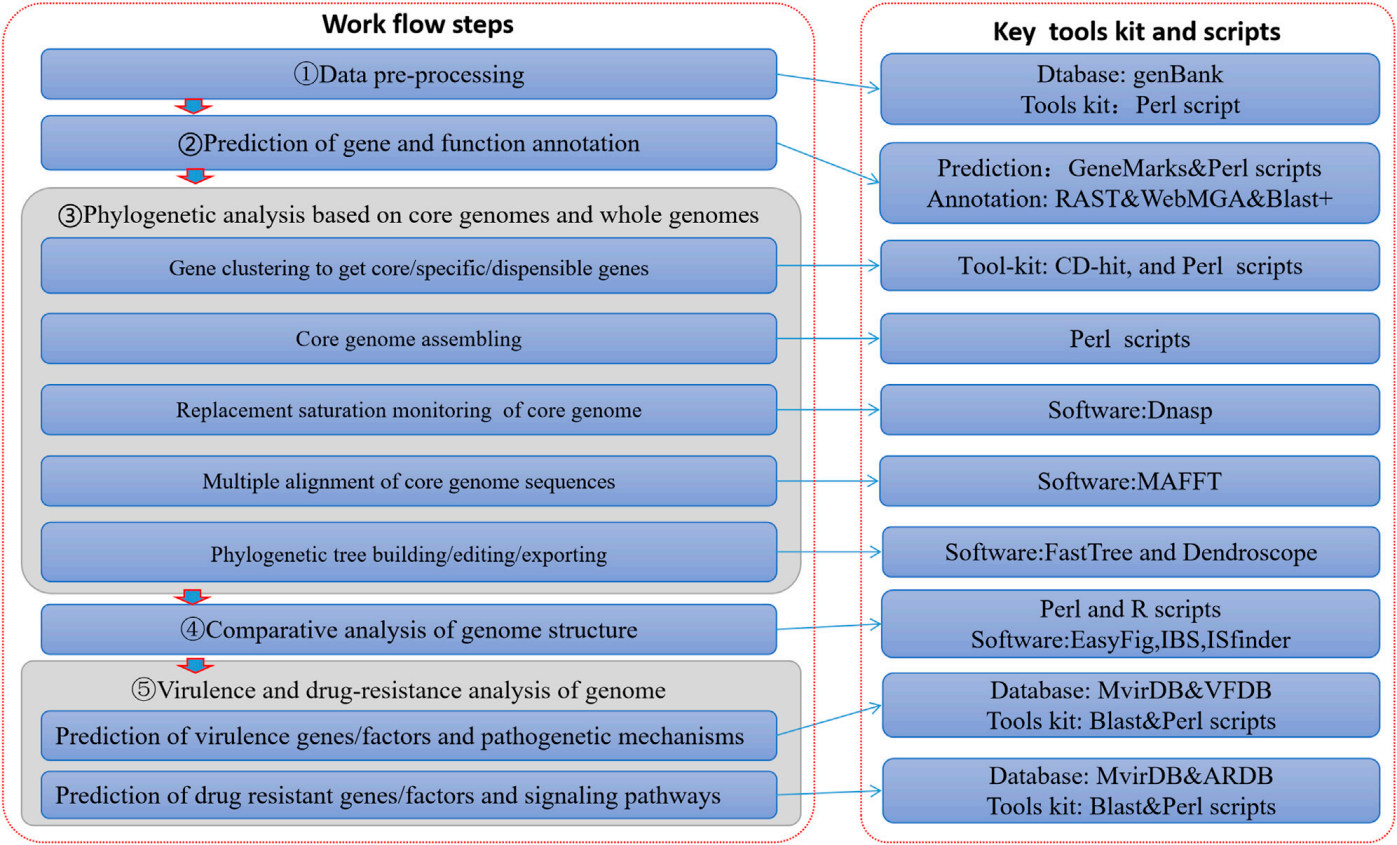
Process framework of infectious pathogen genomes with visualization analysis.

At present, many deficiencies exist in relevant studies: studies on just one specific genus or species of pathogenic bacteria (Ang et al., 2014; Choo et al., 2014a; Choo et al., 2014b; Heydari et al., 2014a; Heydari et al., 2014b; Tan et al., 2015), or studies focusing totally on micro-organisms (such as bacteria) (Marcos et al., 2006; Uchiyama, 2007). So thus far, the depth and precision of data mining has not met the tailored needs of scientific research and clinical practice. In order to solve these problems, this paper constructed a visualization process framework for genome information based on big data mining technology (Figure 1). This process not only covers the comparative genome research of multiple infectious disease pathogens, but also has the ability to mine pathogen genome data deeply. It can therefore accommodate the depth and breadth of pathogens required for molecular level research.

Based on intra-species genome comparisons, a series of analyses was carried out, such as data mining and advanced visual representation of genome information. We studied pathogen genome data to investigate topics such as classification of intra-genera and intra-species, phylogenetic evolution, genome structure, virulence factors (related to pathogenic risk) and resistance factor (related to drug-resistance) carried in genomes and so on. We attempted to determine these characteristics at the molecular level to provide methods for rapid identification of new or unknown pathogens. The correlation research between genome structure differences and biological phenotype characteristics can provide a basis for guidelines of clinical medication and infection control.

## Materials and Methods

On the basis of genome-wide data of pathogens, which can cause communicable diseases, an analysis process framework was built through to allow intra-genera and intra-species comparative genomics research with the integration of a series of algorithm toolkits. It was designed as an information visualization analysis process framework based on large data mining of pathogen genomes (Figure 1). This framework realized a series of functions such as prediction and annotation of genomes, analysis of phylogenetic evolution, comparison of genome structures, and prediction of virulence and resistance factors carried in the genomes. This framework consisted of five data processing steps (Figure 1), detailed as follows:

### Data Preprocessing

The aim of data preprocessing was to meet the format requirements of input files in the subsequent steps of data processing and analysis. Scripts of Perl language (5.0) were used to extract the key description information from source raw data and to convert it into FASTA format. The source raw data of bacteria genomes were downloaded from genomes database of NCBI (http://ftp.ncbi.nih.gov/genomes/Bacteria/) and pubmlst (http://pubmlst.org/neiseria/seqbin/ID). The genome data of human papilloma virus were from GenBank (http://www.ncbi.nih.gov/nucleotide/SRA/genBank). All of these raw data files were in formats of FNA, GBK and EMBL format.

### Prediction of Gene and Function Annotation

The full-length genome sequences in FASTA format above were used as input files to RAST toolkits (Overbeek et al., 2014), GeneMarks (Besemer et al., 2001) and Perl scripts to predict genes. Intergenic DNA was eliminated from the full-length genome sequence. Coding sequences (CDS) were extracted including nucleotide sequence and corresponding protein sequence of the gene. All of these CDS were input into Blast+ (Altschul et al., 1990), KOBAS (Wu et al., 2006), and WebMGA (Wu et al., 2011) to conduct homologous comparison searches and to annotate the function of genes. The annotated files could then be used for correlation research between genome structure and biological or functional characteristics in subsequent steps.

Many public biological databases are referenced in the gene function annotation process, such as NT (genetic information database of all species: https://ftp.ncbi.nlm.nih.gov/blast/db/nt.* tar in gz), Swiss-PROT (protein sequence database with annotation information: https://ftp.ncbi.nlm.nih.gov/blast/db/swissprot.tar.gz), KEGG (signaling pathways and metabolites: https://www.genome.jp/kegg/) and COG (classification database of homologous protein comments: https://www.ncbi.nlm.nih.gov/COG/).

### Phylogenetic Analysis Based on Core and Whole Genomes

Phylogenetic analysis was carried out to analyze the differences between intra-species pathogen genomes. The purpose of genome comparison was to explore the relationship between identification and phylogenetic evolution. In turn, pathogenicity prediction can be given as high risk, low risk and potential risk. It will become an auxiliary method for the classification and identification of new or unknown pathogens. The process of phylogenetic analysis included five parts. One part was about gene clustering to get core/specific/dispensible genes. The coding sequence obtained in step 2) was used as input data for the CD-HIT toolkit (Fu et al., 2012) for genetic clustering analysis and genome comparison. Gene orthologs were clustered and determined by CD-HIT with a nucleotide sequence identity threshold of 0.7 and *n*-word parameter of 4. Each cluster had one representative gene sequence. A matrix describing genomic differences was constructed using Perl script based on CD-HIT cluster results. The core genes were obtained intra-genus or -species from pathogen genomes. Core genes are shared by all reference genomes, while specific genes only exist in one reference genome but are absent from all others. Dispensable genes must be shared in some reference genomes but not all genomes. Collectively, the above three kinds of genes are called pan-genome. The pan-genome was converted to a matrix of genomic differences by Perl scripts. The pan-genome Venn diagram was drawn with the ggplot2 algorithm of R2.4.1. The pan-genome heatmap was built based on the matrix of genomic differences to realize the intuitive visualization for gene distribution differences. Another part was about core genome assembling. The total core gene sequences were assembled according to the original location in the chromosome through Perl scripts to set up the core genome. A third part was about replacement saturation monitoring of the core genome. DnaSP software (Rozas, 2009) was used to evaluate replacement saturation of the core-genome sequence. If the mean of Ka-Ks was far less than 1 (unsaturated), then the genome was suitable for construction of a phylogenetic tree. Another part was about multiple alignment of core genome sequences. Multi-sequence alignment of the core genomes was carried out with MAFFT software (Katoh and Standley, 2013). The other part was about phylogenetic tree building, editing and exporting. Availability of core and whole genomes made it possible to construct phylogenetic trees in this study using FastTree (Price et al., 2009) with an approximate maximum likelihood algorithm. The phylogenetic trees of the core and whole genomes were inferred and edited with Dendroscope 3 (Huson and Scornavacca, 2012), clearly and visually.

### Comparative Analysis of Genome Structures

In this study, we tried to find relationships between the structural basis and the biological characteristic phenotypes of strains at the molecular level. On the basis of the above analysis of gene distribution and genomic phylogenetic relationship, it was necessary to conduct a comparative analysis of the internal structure of the genome for pathogenic genomic differences. For that reason, the parallel comparison diagram of genome structures was drawn by Easyfig (Sullivan et al., 2011), IBS (Liu et al., 2015) and Perl scripts. All input files for this step were the nucleotide sequences annotated through step 2. The structural differences between genomes were shown, such as insertion sequences and deletions.

### Virulence and Drug-Resistance Analysis

The prediction of virulence genes/factors and pathogenetic mechanism was carried out based on two virulence gene/factor databases (VFDB (Chen et al., 2005) and MirvDB (Zhou et al., 2007)), and we based the prediction of drug resistant genes/factors and signaling pathways on a drug-resistance genes/factor database (ARDB (Liu, 2009)). It was also necessary to research the relationship between the differences in nucleotide level and virulence/drug-resistance of the pathogens. Homologous protein sequences were extracted by Blast+ (Blastp was chosen) program from the virulence genes/factor databases. and the drug-resistance genes/factor database. The protein sequence inputs were also obtained from step 2. Important information was obtained accordingly, such as regulation-control systems, pathogenic mechanisms of virulence genes/factors, and the signaling pathways of the drug resistant genes/factors. Results were visualized using the ggplot2 and heatmap packages of R language. The different distribution and density of the virulence/resistance factors can be intuitively shown by using gradual or contrasting colors. All of the results helped us to understand intuitively the distribution and existence modes of all virulence and resistance factors in all genomes, and to understand the relationship between genetic differences and properties of virulence and resistance.

## Results

This section is mainly in view of the relationship between genomic differences and biology phenotypic characteristics in accordance with the above visualization process framework. Data preprocessing laid a good foundation for gene prediction and functional annotation. Phylogeny is closely related to gene distribution. Characteristics of virulence and drug-resistance are also closely associated with genomic differences. The experiments were carried out from three angles: phylogeny (macro perspective), structure differences of genomes (micro perspective), and virulence and drug-resistance characteristics (prediction perspective). The visual analysis results show that the process framework presented in this paper is applicable to general research of disease prevention and control of pathogenic organisms. The framework can help to recommend the most appropriate strains for clinical and scientific research.

### Gene Prediction and Function Annotation

The role of gene prediction was to extract coding and protein sequences from full-length genomes, while function annotation was to add biological function information to nucleotide sequences obtained by gene prediction.

The bacterial genomic big data obtained for this study included genomes from four genera: 44 reference strains of Fusobacterium, 67 reference strains of Streptococcus, 51 reference strains of Neisseria and 44 reference strains of Streptococcus salivarius. Taking the 44 strains of Streptococcus salivarius as an example, the whole genome data was pre-processed by format transformation, and then, input into RAST and GeneMarks toolkits to complete gene prediction and obtain the coding sequences and corresponding protein sequences (FASTA format). On this basis, the tools recommended in the process framework were used to carry out gene function annotation. Function annotation can provide information for subsequent studies on the correlation between gene function and biological phenotypic characteristics, as detailed in the following subsections. There were three steps for gene function annotation as follows: 1) the nucleotide sequence was input into the Blast+ program (Blastn was chosen) to search the nucleotide sequences against the NT database and the genome of the ATCG8618 international reference strain, which is fully annotated; 2) the protein sequence was input into the Blast+ program (Blastp was chosen) against the Swiss-PROT database to fetch homologous protein sequences and related annotation information; 3) the protein sequences were input into KOBAS and WebMGA software simultaneously to search for KEGG pathway and COG annotation information. Recommended values were used for Blastn parameter thresholds (Hogg et al., 2007; Laing et al., 2010) (identity ≥70, coverage ≥70%, evalue <1e−5, score >50) and Blastp parameter thresholds (Lefebure and Stanhope, 2007; Ostlund et al., 2010) (identity ≥30, coverage ≥50%, evalue <1e−5, score > 50).

### Phylogenetic Analysis Based on Core and Whole Genomes

Phylogenetic analysis based on core genomes was used to explore the relationship between gene distribution characteristics and phylogenetic evolution of the core genomes. Gene distribution of the genomes refers to the distribution of homologous core gene clusters, dispensable gene clusters and specific gene clusters obtained by comparison of intra- or inter-species genomes. The core genome was then assembled by ordering the core genes. The core genome phylogenetic analysis refers to building of a phylogenetic tree based on the core genomes of the strains. The phylogenetic tree was used to predict the difference, affinity and potential pathogenicity risk of different subtypes of bacterial strains according to the location of the strain in the tree. It can assist with the identification of a new or unknown pathogen, so as to facilitate the prevention and control of their transmission.

**FIGURE 2:**
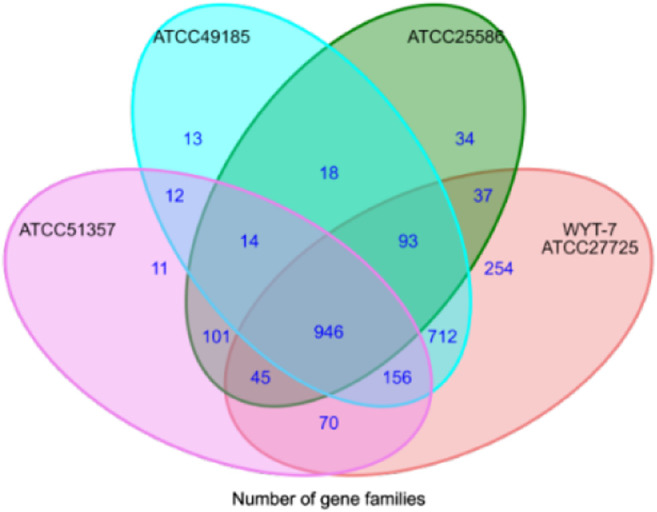
Venn diagram of comparison analysis for Fusobacterium genomes.

Taking 44 strains of Fusobacterium as an example, the nucleotide sequences were input into CD-HIT software (identity *c* = 0.7 and *n* = 5) in accordance with the process framework to conduct homologous clustering for functional genes. We aimed to search for core genes, specific genes and dispensable genes intra- or interspecies. Representative sequences were selected for each gene cluster. For multi-copy sequences, the best-matched gene with the longest length was selected as the representative sequence for the gene. The output data from the CD-HIT software was converted into a difference matrix of gene distribution by Perl scripts. The ggplot2 algorithm package of *R* language was used to draw a Venn diagram to complete the visual display of gene distribution (Figure 2).

**FIGURE 3:**
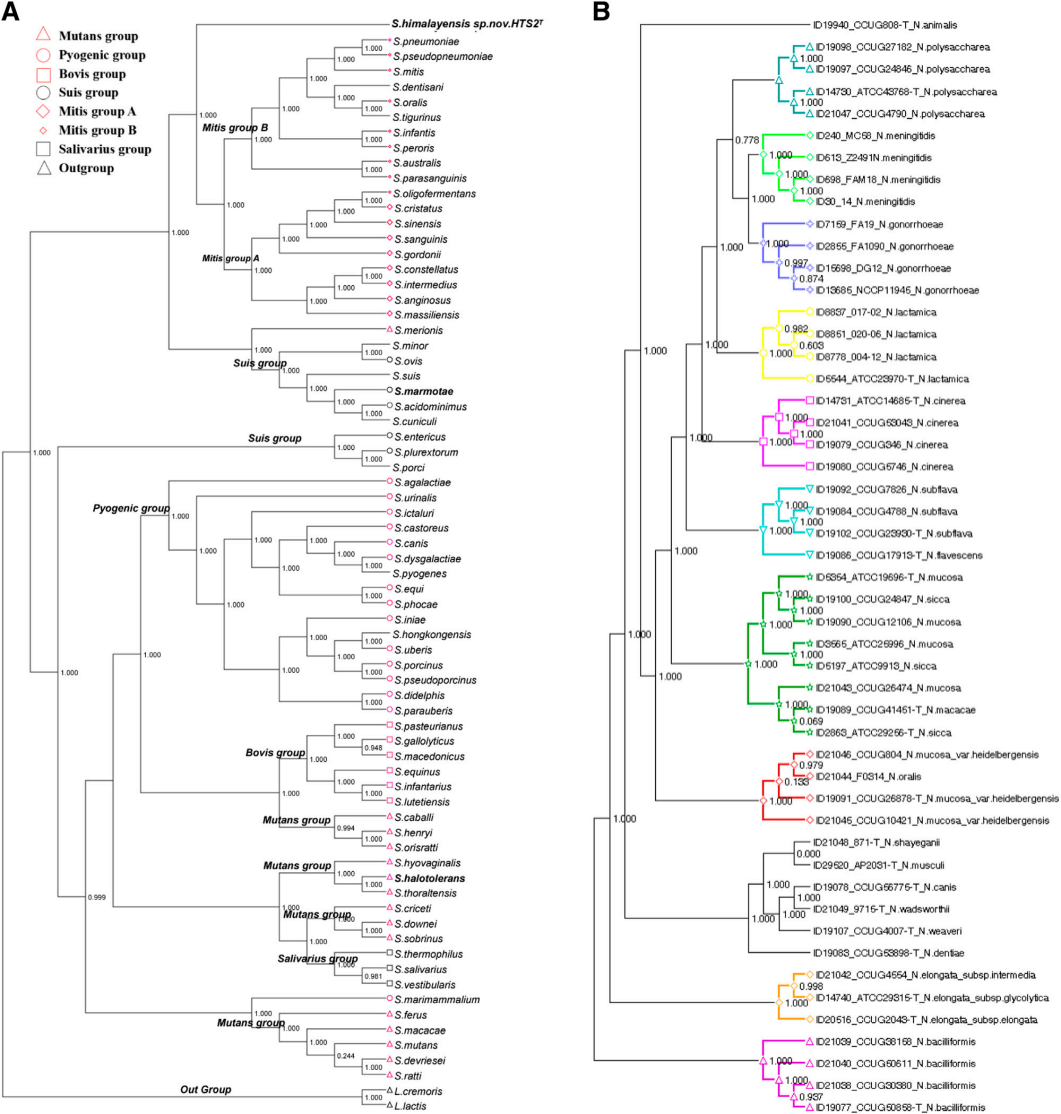
Phylogenetic tree of bacteria core genomes. **(A)** Streptococcus; **(B)** Neisseria.

The core genes were assembled into the core genome sequence according to their location on chromosomes. The mean value of Ka/Ks (synonymous replacement rate) was calculated with DnaSP software for every core genome. If the value was less than 1, it indicated that the nucleotide sequence evolved slowly with unsaturated replacement and was suitable for building phylogenetic trees. To implement relevant data processing, three software of MAFFT, FastTree and Dendroscope were used. In details, MAFFT was used for multi sequence alignment of core genomes, FastTree was used for the calculation of the genetic evolutionary distance between the aligned sequences to construct the phylogenetic tree, and Dendroscope 3.0 was used to visualize the core genome phylogenetic tree (Figure 3).

Pathogens clustered in different clusters according to their genetic evolutionary distances. If they were located in the same cluster and closely with each other in the tree, it suggested that they had evolved from the same ancestor. Accordingly, the following inferences could be made. If a phylogenetic tree of core genomes was built with known pathogens and a new or unknown pathogen, there will be two situations: 1) if the new or unknown pathogen is clustered in one cluster with the known strains, it indicates that the strains are closely related and may have pathogenetic risks similar to the known pathogens; 2) the new bacteria was clustered into independent branches, which could predict that the strain might be a new pathogen. To verify the above inference, we conducted an experiment using the Streptococcus genome. It is not difficult to see from Figure 3A that Streptococcus himalayensis sp. nov.HTS2T was isolated from all other clusters of the Mitis group, and its location in the phylogenetic tree indicates that Streptococcus himalayensis may be a new species. This conclusion was consistent with the results of the 16sRNA phylogenetic tree, DDH online hybridization for strain typing, and with the results of biological validation experiments (Niu et al., 2017). Part of the present method has also made a great contribution to the isolation and identification of three new Streptococcus strains (Niu et al., 2016a; Niu et al., 2016b; Niu et al., 2017) (Streptococcus himalayensis_hts2, Streptococcus halotolerans_hts9, Streptococcus marmotae_hts5) and one strain of Fusobacterium (Wang et al., 2016).

To prove the generality of the method, we applied the method to the Neisseria genome comparison to obtain the core genome for construction of a phylogenetic tree (Figure 3B). The results show that the affinity or pathogenetic risk of pathogens was closely related to the location of genomes in the phylogenetic tree. At the same time, these results of clade distribution in the phylogenetic tree were consistent with the results of phylogenetic trees built respectively by rplF genes and rMLST genes. Neisseria bacterium species isolation or identification is always based on the phylogenetic tree of rplF and rMLST genes (Bennett et al., 2013; Bennett et al., 2014). Therefore, the phylogenetic tree of the core genome in this paper can provide support for the prediction of the relationship between new and the other pathogens and their pathogenetic risk, as well as the basis for identification of new pathogens and prevention and control of infection. Moreover, the experimental results also suggested that the process framework designed in this paper can be extended to the identification of other new pathogens.

### Comparative Analysis of Genome Structure

In this section, we attempted to combine the information regarding gene function annotation to study the correlation between structural differences of genomes and biological phenotypic characteristics of species from the microscopic perspective. We aimed to provide data to support the selection of vaccine strains or excellent strains for practical applications. Taking Streptococcus salivarius as an example, different subtypes of the species demonstrated different deodorant functions. The deodorant function is associated with the number and distribution of specific genes. According to the literature (Hyink et al., 2007), the genome of S. salivarius_K12 (K12) performed a better deodorization function in biological tests compared with other S. salivarius strains due to the inclusion of Sbo and Sal series genes. Therefore, based on the gene function annotation information previously described, the genes with redox-reaction and bacteriocin functions were selected out because of a potential close relationship with the deodorant function of decomposition of hydrogen sulphide. According to the relationship between specific functional genes and biological phenotypic characteristics, practical applications will be guided and evidence will be provided for strain recommendations with better performance of deodorization, such as for oral hygiene products (mouthwash).

**FIGURE 4:**
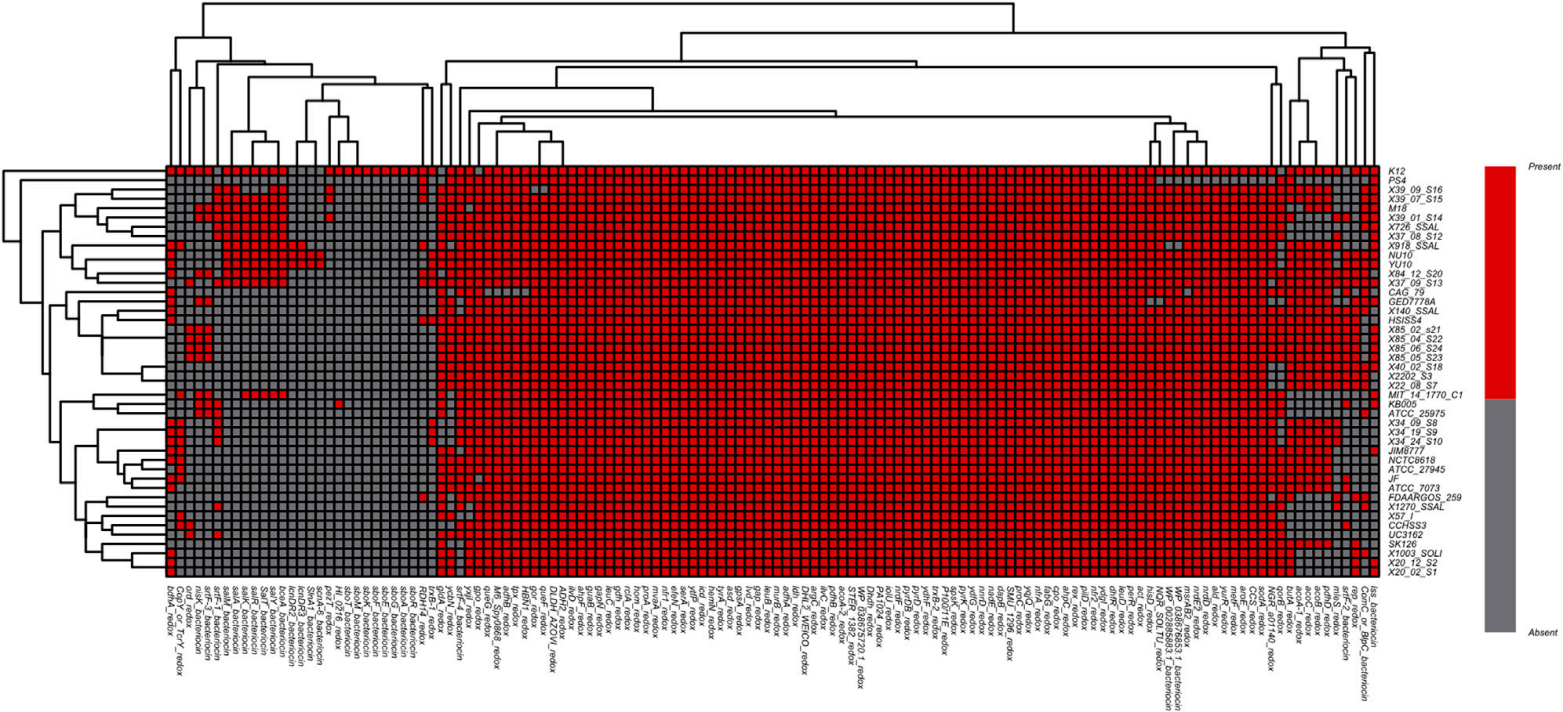
Heatmap of function-specific genes related to redox reaction and bacteriocin among S. salivarius genomes (Red indicates the gene [row] is present in strains [column], and gray indicates the gene [row] is absent in strains [column]).

Following this logic, a comparison study was carried out among all 44 strains of Streptococcus salivarius (including K12) genomes above. The deodorization function was closely related to Sbo and Sal series genes. However, the comparison result showed that the Sbo was unique to the K12 gene clusters. While the cluster of Sal series genes existed in strain genomes such as 39-01-S14, M18, 37-08-S12, 26-SSAL, 84-12-S20, 37-09-S13, YU10, NU10, 918_SSAL and 39-07-S15, the distribution of genes related to redox-reaction or bacteriocin function was also different in each genome (Figure 4).

**FIGURE 5:**
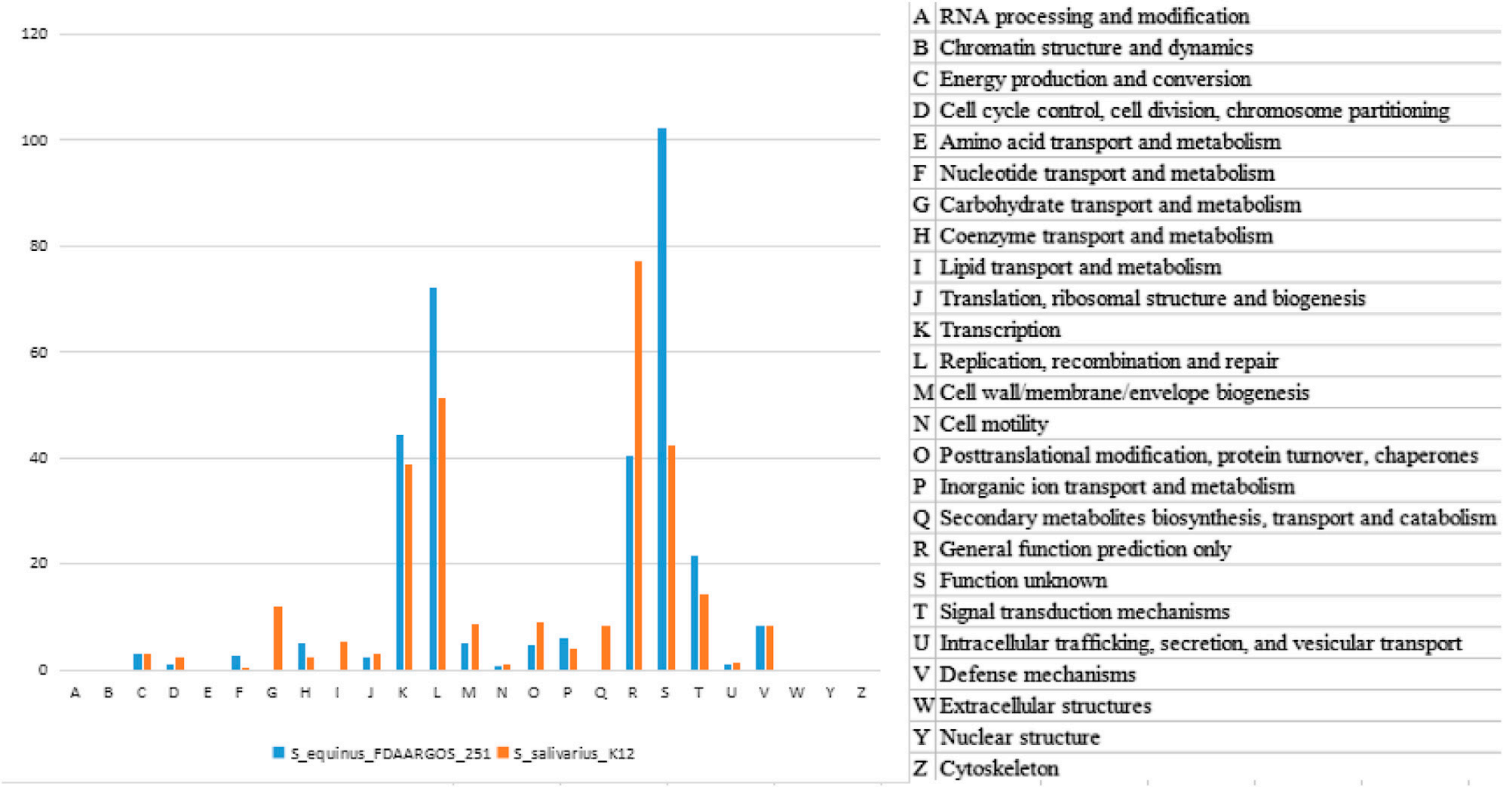
Gene function category of plasmid genomes between K12 and 251 from the COG database annotation.

The two clusters of K12 specific genes Sbo and Sal were located on the plasmid carried by K12. We therefore traced the K12 plasmid genome in the NT database to find the original source of the Sbo genes. The sequence with the highest similarity (identity 98%, coverage 56%) was obtained from the plasmid carried by S.equinus_251 (251). In this paper, two plasmid sequences of K12 and 251 were searched for homologous sequences in the COG database. This process was called homologous protein annotation classification (Figure 5). It was found that the functional classification and distribution trends of genes on the two plasmids of K12 and 251 were roughly the same, and the G type and Q type genes of K12 were unique. This unique classification may give rise to more new function-specific proteins, suggesting that K12 may have different biological characteristics.

**FIGURE 6:**
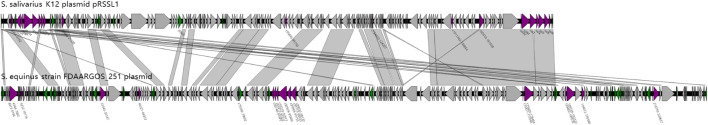
Parallel comparison diagram between the genome structure of K12 and 251 with specific functional genes in the whole plasmid genome (purple triangles indicate the specific genes have deodorization function. Green triangles indicate insertion sequences (IS). Gray shading indicates similarity of more than 70% between the two plasmid genomes).

A further comparison was carried out between the two plasmid genome structures based on the functional annotation. The result was shown as a parallel comparison diagram using Easyfig software (Figure 6). It can be seen that the skeletons of plasmids carried respectively by 251 and K12 had high similarity (identity above 70%) with each other. Among them, Sal genes of the K12 plasmid had highly homologous areas of gene clusters closely related to deodorization function in the plasmid of 251 strain, but the Sbo cluster did not exist in this plasmid.

**FIGURE 7:**
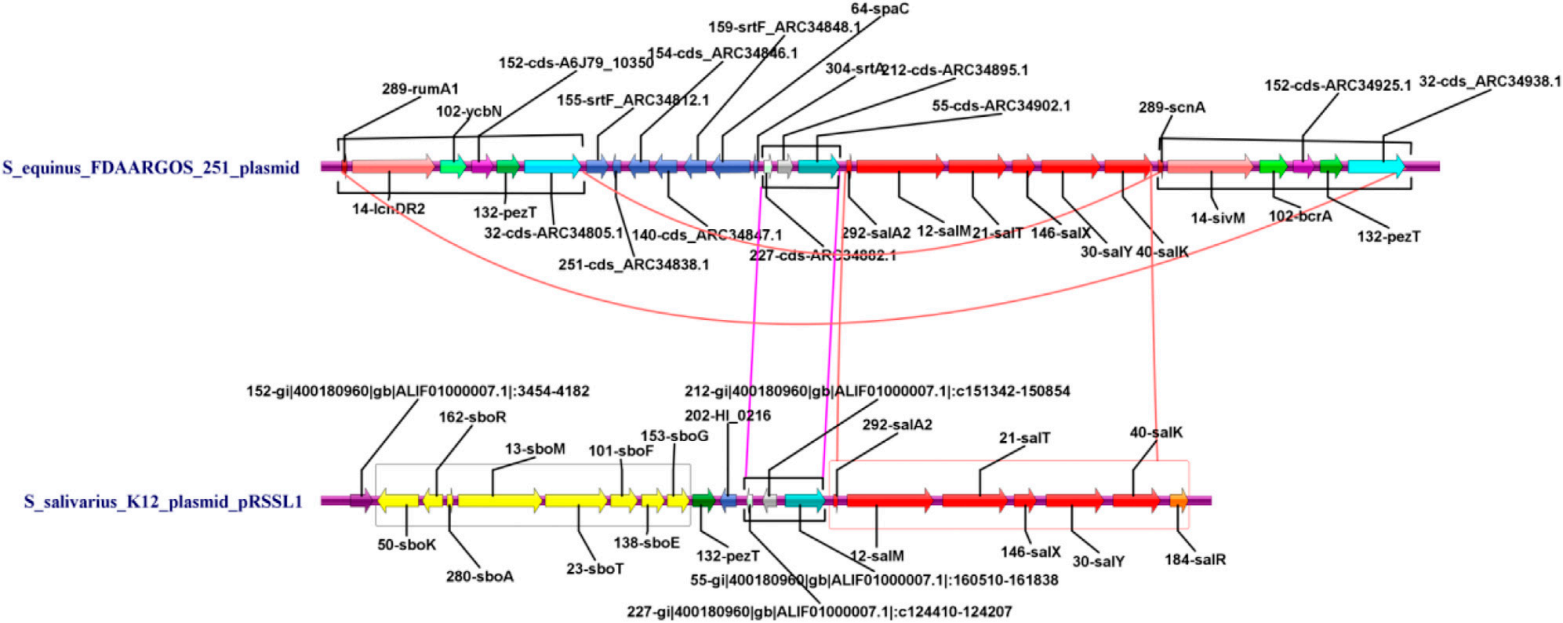
Parallel comparison diagram of genome structure with specific functional genes.

All genes associated with the deodorization function (such as bacteriocin and redox-reaction function) were obtained by combining the gene functional annotation information. They were assembled together according to their inherent order, strand, and length in the chromosome. A parallel comparison diagram of the genome structure was drawn using IBS software with these specific functional genes to reflect their distribution (Figure 7). It can be seen from Figure 7 that the K12 plasmid genome contained Sbo series genes (highlighted in yellow), while the plasmid of 251 did not have these specific genes. The K12 plasmid had seven Sal genes (red) while the 251 plasmid had only six (red, SalR gene is absent). The K12 strain carrying the plasmid had strong deodorization ability, because it contained Sbo and Sal series genes, especially Sbo genes. Therefore, the K12 strain carrying the plasmid can be used to produce oral hygiene products such as mouthwash (Hyink et al., 2007). These results provide molecular evidence for biological characteristics. In addition, the plasmid of 251 also had a large area of repeat sequence in a consistent order (Figure 7, connected with two magenta lines), and the Sal genes of 251 had the same function as the K12 plasmid (Figure 7, red arrows).

### Virulence and drug resistance analysis of genomes

Pathogens manifest their pathogenicity and drug resistance through the expression of virulence and drug resistance genes. The expression patterns of these genes and factors are expressed at the levels of genes and biochemical or structural characteristics. The resulting pattern can increase strains’ pathogenetic risk or likelihood of drug resistance by acting either internally or directly on the host. The acquisition of a single virulence or drug resistance factor may convert a non-pathogenic strain to a pathogenic strain, or may render a sensitive strain drug-resistant. The more virulence genes or factors carried by a given strain, the higher their potential pathogenetic risk may be. Therefore, it is very import to identify virulence or drug resistance factors. The identification and verification of virulence gene characteristics often rely on comparative pathogenomic approaches, which generally reveal the nature and phylogeny of virulence and resistance. It will also enable new therapies or preventive measures to become possible.

**FIGURE 8:**
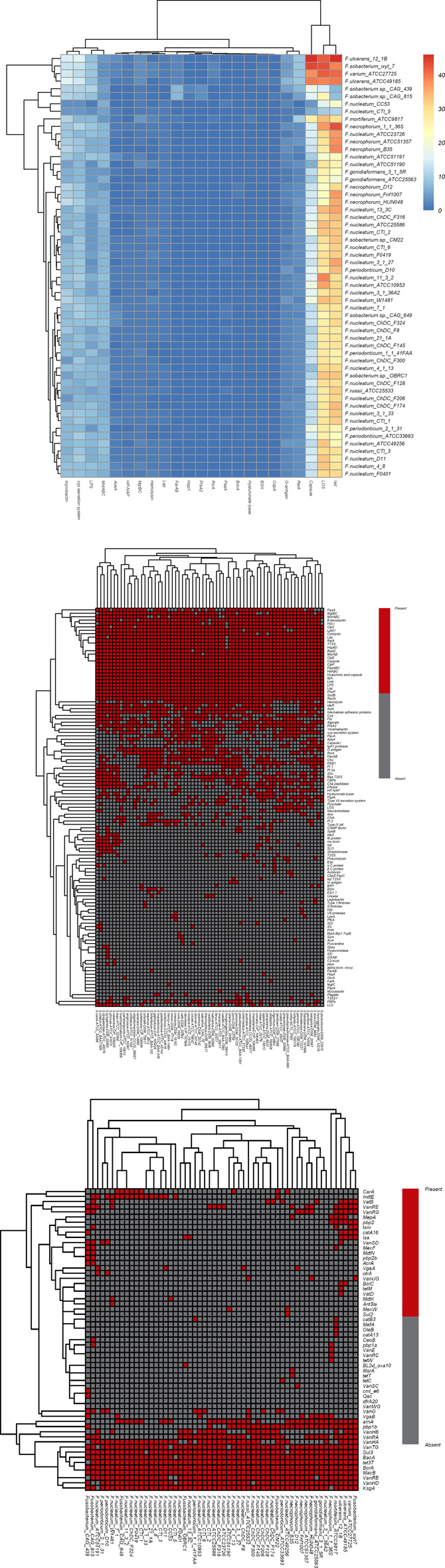
Heatmaps to show the virulence or drug resistance factors. **(A)** Heatmap of virulence factors in Fusobacterium genomes; **(B)** heatmap of virulence genes in Streptococcus genomes; **(C)** heatmap of drug resistance factors in Fusobacterium genomes. For **(A)**, The color changes from blue to red with increasing numbers of virulence factors; For **(B and C)**, red indicates the gene [row] is present in strains [column], and gray indicates the gene [row] is absent in strains [column]).

In this study, the amino acid sequences of 44 strains of Fusobacterium and 67 strains of Streptococcus were used as test data sets. Homology comparisons were performed by Blastp software against VFDB and MvirDB databases (virulence genes/factors) to predict virulence gene/factor information and also related pathogenic mechanisms (Figures 8A,B).

Homology comparisons were performed by Blastp software against the ARDB database to predict drug resistance factors and related metabolic pathways (Figure 8C). The ggplot2 algorithm package of R language was used for visualization of the prediction results, and the presence of virulence genes in each pathogen genome was demonstrated by a clustering heatmap of virulence genes/factors. In the above processing, Blastp software parameters were set as the recommended values (24,25) (e-value ≤ 1e−5, identity ≥30%, query coverage ≥50%, score ≥50).

It can be seen from Figure 8 that the pattern of virulence genes/factors determined the location of the strain in the cluster tree. In Figure 8A, the four strains (Fusobacterium_wyt_7, Fusobacterium ulcerans_12_1B, Fusobacterium varium_ATCC27725 and Fusobacterium ulcerans_ATCC49185) include the largest number of virulence genes belonging to three virulence factors (LOS(lipoligosaccharide), capsule and Isd(iron-regulated surface determinant)), indicating that virulence characteristics of the four strains may be consistent with each other. As the virulence genes and factors carried by these strains increase, their potential pathogenetic risk may also increase.

In addition, the heatmap (Figure 8B) of virulence genes in Streptococcus showed there were more virulence genes/factors in S.agalactiae-ATCC_13813 (52 genes/factors), S.iniae-ATCC_29178 (52 genes/factors), S.thoraltensis-DSM_12221 (52 genes/factors), S.pseudoporcinus-ATCC_BAA-1381 (53 genes/factors), S.canis-ATCC_43496 (52 genes/factors), S.castoreus-CIP_108205 (53 genes/factors), S.equi-ATCC_33398 (54 genes/factors), S.dysgalactiae-ATCC_43078 (56 genes/factors), S.pyogenes-DSM_20565 (57 genes/factors) and S.ictaluri-ATCC_BAA-1300 (59 genes/factors). These ten strains are all belong to the pyogenic A Group, meaning that the biological phenotypic characteristics of these strains are consistent with each other. The phylogenetic tree (Figures 8A,B) constructed according to the distribution pattern of virulence genes/factors was broadly consistent with the phylogenetic tree constructed according to the distribution pattern of core genomes (Niu et al., 2016a; Niu et al., 2016b; Wang et al., 2016; Niu et al., 2017) . This indicates that virulence genes/factors may be closely related to core genes. In other words, virulence genes are also key genes in the evolutionary differentiation of species. It suggests that specific virulence genes may contribute to differing pathogenetic risks of strains. The information from these virulence genes/factors may provide clues and evidence for biological experiments regarding pathogenesis. The drug resistance factors and related pathway information for Fusobacterium were also predicted (Figure 8C) against ARDB (drug-resistance genes/factors), which could provide clues to the molecular information related to drug resistance in Fusobacterium .

## Discussion

This paper presents a novel genome information visualization analysis framework based on big data mining technology. Different from the existing related researches which mostly focused on a specific genus, a specific species or a single function, the framework presented in this paper fully considers both depth and precision for pathogen genomes and hence can be generalized for analysis of many bacterial genomes. It can meet the requirements for scientific research and clinical practice for at least four different kinds of pathogen identification and classification. Part of the process framework has made great contribution to species identification and analysis of Streptococcus, Fusobacterium and Human papilloma virus (Chen et al., 2020a) based on genome big data mining. It provided the necessary evidence for the rapid confirmation of new or unknown HPV strain based on the workflow of this paper, which can theoretically support the early prevention and diagnosis of clinical cervical cancer.

It has been applied to a series of pathogen genome analyses, including three genera (Fusobacterium, Streptococcus, and Neisseria) and one species (Streptococcus salivarius) in this paper. The process framework can achieve genome-wide big data mining for a variety of pathogenic bacteria by supporting multi-level and multi-angle analysis.

This framework includes five functions, they are genome annotation, phylogeny analysis based on core genome, analysis of structure differences between genomes, prediction of virulence genes/factors with their pathogenic mechanisms, and prediction of resistance genes/factors with their signaling pathways. It can undoubtedly provide data support for identification of new pathogens quickly and contribute to prevention and control of the pathogen.

During the genome information analysis, some existing tools, software and algorithm packages were flexibly applied to realize a diversified display of results of genome big data analysis (Cheng, 2019; Cheng et al., 2019; Yang et al., 2019; Dao et al., 2020a; Dao et al., 2020b; Wu et al., 2020; Zhang et al., 2020), so as to facilitate intuitive and quick understanding of the molecular structure characteristics behind different biological phenotypic characteristics as well as possible correlations between them.

## Data Availability

The original contributions presented in the study are included in the article/Supplementary Material, further inquiries can be directed to the corresponding authors.

## Abbreviations

TB, Tuberculosis bacilli; CDS, Coding sequences; LOS, lipoligosaccharide; Isd, iron-regulated surface determinant.

## References

[B1] Altschul S. F. Gish W. Miller W. Myers E. W. Lipman D. J. (1990). Basic local alignment search tool. J. Mol. Biol. 215 (3), 403–410. 10.1016/S0022-2836(05)80360-2 2231712

[B2] Ang M. Y. Heydari H. Jakubovics N. S. Mahmud M. I. Dutta A. Wee W. Y. (2014). FusoBase: an online Fusobacterium comparative genomic analysis platform. Database (Oxford) 2014 (239), 148. 10.1093/database/bau082 PMC4141642 25149689

[B3] Bennett J. S. Jolley K. A. Maiden M. C. J. (2013). Genome sequence analyses show that Neisseria oralis is the same species as 'Neisseria mucosa var. heidelbergensis'. Int. J. Syst. Evol. Microbiol. 63 (Pt 10), 3920–3926. 10.1099/ijs.0.052431-0 24097834 PMC3799226

[B4] Bennett J. S. Watkins E. R. Jolley K. A. Harrison O. B. Maiden M. C. (2014). Identifying Neisseria species by use of the 50S ribosomal protein L6 (rplF) gene. J. Clin. Microbiol. 52 (5), 1375–1381. 10.1128/JCM.03529-13 24523465 PMC3993661

[B5] Besemer J. Lomsadze A. Borodovsky M. (2001). GeneMarkS: a self-training method for prediction of gene starts in microbial genomes. Implications for finding sequence motifs in regulatory regions. Nucleic Acids Res *.* 29 (12), 2607–2618. 10.1093/nar/29.12.2607 11410670 PMC55746

[B6] Chen C. X. Cao Z. F. Li T. J. Yu L. Yu Y. F. Cai R. K. (2020a). Genome research analysis for human papilloma virus based on big-data mining and visualization analysis. J. Reprod. Med. 29 (10), 1362–1368. 10.3969/j.issn.1004-3845.2020.10.019

[B7] Chen C. X. Wang X. L. Jiang T. J. Cao Z. F. Li T. J. Yu L. (2020b). Platform construction for the early-warning forecast in prevention and control of influenza based on multi-source heterogeneous big-data mining. China Biotech. 40, 109–115. 10.13523/j.cb.1906028

[B8] Chen L. Yang J. Yu J. Yao Z. Sun L. Shen Y. (2005). VFDB: a reference database for bacterial virulence factors. Nucleic Acids Res *.* 33 (Database issue), D325–D328. 10.1093/nar/gki008 15608208 PMC539962

[B9] Cheng L. (2019). Computational and biological methods for gene therapy. Curr. Gene. Ther. 19 (4), 210. 10.2174/156652321904191022113307 31762421

[B10] Cheng L. Zhao H. Wang P. Zhou W. Luo M. Li T. (2019). Computational methods for identifying similar diseases. Mol. Ther. Nucleic Acids 18, 590–604. 10.1016/j.omtn.2019.09.019 31678735 PMC6838934

[B11] Choo S. W. Ang M. Y. Fouladi H. Tan S. Y. Siow C. C. Mutha N. V. (2014a). HelicoBase: a helicobacter genomic resource and analysis platform. BMC Genomics 15 (1), 600. 10.1186/1471-2164-15-600 25030426 PMC4108788

[B12] Choo S. W. Heydari H. Tan T. K. Siow C. C. Beh C. Y. Wee W. Y. (2014b). VibrioBase: a model for next-generation genome and annotation database development. Sci. World J. 2014, 569324. 10.1155/2014/569324 PMC4138799 25243218

[B13] Cole S. T. (2002). Comparative mycobacterial genomics as a tool for drug target and antigen discovery. Eur. Respir. J. Suppl. 36, 78s–86s. 10.1183/09031936.02.00400202 12168750

[B14] Dao F. Y. Lv H. Yang Y. H. Zulfiqar H. Gao H. Lin H. (2020a). Computational identification of N6-methyladenosine sites in multiple tissues of mammals. Comput. Struct. Biotechnol. J. 18, 1084–1091. 10.1016/j.csbj.2020.04.015 32435427 PMC7229270

[B15] Dao F. Y. Lv H. Zulfiqar H. Yang H. Su W. Gao H. (2020b). A computational platform to identify origins of replication sites in eukaryotes. Brief Bioinform. 0 (0), 1–11. 10.1093/bib/bbaa017 32065211

[B16] Fu L. Niu B. Zhu Z. Wu S. Li W. (2012). CD-HIT: accelerated for clustering the next-generation sequencing data. Bioinform. 28 (23), 3150–3152. 10.1093/bioinformatics/bts565 PMC3516142 23060610

[B17] Gilmour M. W. Graham M. Reimer A. Van Domselaar G. (2013). Public health genomics and the new molecular epidemiology of bacterial pathogens. Public Health Genomics 16 (1-2), 25–30. 10.1159/000342709 23548714

[B18] Heydari H. Mutha N. V. Mahmud M. I. Siow C. C. Wee W. Y. Wong G. J. (2014a). StaphyloBase: a specialized genomic resource for the staphylococcal research community. Database (Oxford) 2014 (1), bau010. 10.1093/database/bau010 24578355 PMC5630900

[B19] Heydari H. Siow C. C. Tan M. F. Jakubovics N. S. Wee W. Y. Mutha N. V. (2014b). CoryneBase: corynebacterium genomic resources and analysis tools at your fingertips. PLoS One 9 (1), e86318. 10.1371/journal.pone.0086318 24466021 PMC3895029

[B20] Hogg J. S. Hu F. Z. Janto B. Boissy R. Hayes J. Keefe R. (2007). Characterization and modeling of the Haemophilus influenzae core and supragenomes based on the complete genomic sequences of Rd and 12 clinical nontypeable strains. Genome. Biol. 8 (6), R103. 10.1186/gb-2007-8-6-r103 17550610 PMC2394751

[B21] Huson D. H. Scornavacca C. (2012). Dendroscope 3: an interactive tool for rooted phylogenetic trees and networks. Syst. Biol. 61 (6), 1061–1067. 10.1093/sysbio/sys062 22780991

[B22] Hyink O. Wescombe P. A. Upton M. Ragland N. Burton J. P. Tagg J. R. (2007). Salivaricin A2 and the novel lantibiotic salivaricin B are encoded at adjacent loci on a 190-kilobase transmissible megaplasmid in the oral probiotic strain Streptococcus salivarius K12. Appl. Environ. Microbiol. 73 (4), 1107–1113. 10.1128/AEM.02265-06 17194838 PMC1828679

[B23] Kan B. Zhou H. Du P. Zhang W. Lu X. Qin T. (2018). Transforming bacterial disease surveillance and investigation using whole-genome sequence to probe the trace. Front. Med. 12 (1), 23–33. 10.1007/s11684-017-0607-7 29318441

[B24] Karesh W. B. Dobson A. Lloyd-Smith J. O. Lubroth J. Dixon M. A. Bennett M. (2012). Ecology of zoonoses: natural and unnatural histories. Lancet 380 (9857), 1936–1945. 10.1016/S0140-6736(12)61678-X 23200502 PMC7138068

[B25] Katoh K. Standley D. M. (2013). MAFFT multiple sequence alignment software version 7: improvements in performance and usability. Mol. Biol. Evol. 30 (4), 772–780. 10.1093/molbev/mst010 23329690 PMC3603318

[B26] Laing C. Buchanan C. Taboada E. N. Zhang Y. Kropinski A. Villegas A. (2010). Pan-genome sequence analysis using Panseq: an online tool for the rapid analysis of core and accessory genomic regions. BMC Bioinform. 11, 461. 10.1186/1471-2105-11-461 PMC2949892 20843356

[B27] Lefebure T. Stanhope M. J. (2007). Evolution of the core and pan-genome of Streptococcus: positive selection, recombination, and genome composition. Genome Biol. 8 (5), R71. 10.1186/gb-2007-8-5-r71 17475002 PMC1929146

[B28] Liu B. P. M. (2009). ARDB-antibiotic resistance genes database. Nucleic Acids Res. 37 (Database issue), D443–D447. 10.1093/nar/gkn656 18832362 PMC2686595

[B29] Liu W. Xie Y. Ma J. Luo X. Nie P. Zuo Z. (2015). IBS: an illustrator for the presentation and visualization of biological sequences. Bioinform. 31 (20), 3359–3361. 10.1093/bioinformatics/btv362 PMC4595897 26069263

[B30] Mao Ping H. D. Wang Y. H. (2017). Big data analysis of status and trends of global change research. J. Univ. Chin. Acad. Sci. 34 (4), 11. 10.7523/j.issn.2095-6134.2017.04.006

[B31] Marcos C. Mascarenhas D. Degrave D. Basílio de Miranda A. (2006). GenoMycDB: a database for comparative analysis of mycobacterial genes and genomes. Genet Mol. Res. 5 (1), 115–126. 10.1590/S1415-47572006000200033 16755503

[B32] Niu L. Lu S. Hu S. Jin D. Lai X. Yang J. (2016a). Streptococcus halotolerans sp. nov. isolated from the respiratory tract of Marmota himalayana in Qinghai-Tibet Plateau of China. Int. J. Syst. Evol. Microbiol. 66 (10), 4211–4217. 10.1099/ijsem.0.001337 27469933

[B33] Niu L. Lu S. Hu S. Jin D. Lai X. Yang J. (2016b). Streptococcusmarmotae sp. nov., isolated from the respiratory tract of Marmota himalayana. Int. J. Syst. Evol. Microbiol. 66 (11), 4315–4322. 10.1099/ijsem.0.001350 27473166

[B34] Niu L. Lu S. Lai X. H. Hu S. Chen C. Zhang G. (2017). Streptococcus himalayensis sp. nov., isolated from the respiratory tract of Marmota himalayana. Int. J. Syst. Evol. Microbiol. 67 (2), 256–261. 10.1099/ijsem.0.001609 27902227

[B35] Ostlund G. Schmitt T. Forslund K. Kostler T. Messina D. N. Roopra S. (2010). InParanoid 7: new algorithms and tools for eukaryotic orthology analysis. Nucleic Acids Res *.* 38 (Database issue), D196–D203. 10.1093/nar/gkp931 19892828 PMC2808972

[B36] Overbeek R. Olson R. Pusch G. D. Olsen G. J. Davis J. J. Disz T. (2014). The SEED and the rapid annotation of microbial genomes using subsystems technology (RAST). Nucleic Acids Res *.* 42 (Database issue), D206–D214. 10.1093/nar/gkt1226 24293654 PMC3965101

[B37] Price M. N. Dehal P. S. Arkin A. P. (2009). FastTree: computing large minimum evolution trees with profiles instead of a distance matrix. Mol. Biol. Evol. 26 (7), 1641–1650. 10.1093/molbev/msp077 19377059 PMC2693737

[B38] Rozas J. (2009). DNA sequence polymorphism analysis using DnaSP. Methods Mol. Biol. 537, 337–350. 10.1007/978-1-59745-251-9_17 19378153

[B39] Sullivan M. J. Petty N. K. Beatson S. A. (2011). Easyfig: a genome comparison visualizer. Bioinform. 27 (7), 1009–1010. 10.1093/bioinformatics/btr039 PMC3065679 21278367

[B40] Tan S. Y. Dutta A. Jakubovics N. S. Ang M. Y. Siow C. C. Mutha N. V. (2015). YersiniaBase: a genomic resource and analysis platform for comparative analysis of Yersinia. BMC Bioinform. 16, 9. 10.1186/s12859-014-0422-y PMC4384384 25591325

[B41] Uchiyama I. (2007). MBGD: a platform for microbial comparative genomics based on the automated construction of orthologous groups. Nucleic Acids Res. 35 (Database issue), D343–D346. 10.1093/nar/gkl978 17135196 PMC1751538

[B42] Wang Y. T. Dong J. Jing Y. Shan L. Ji P. Xiang-li M. (2016). Isolation and antibiotic resistance detection of Enterococcus gallinarums from Marmota himalayana. Disease Surveillance 31 (5), 7. 10.3784/j.issn.1003-9961.2016.05.008

[B43] Wu C. L.Q. Xing R. Fan G. L. (2020). Using the chou’s pseudo component to predict the ncRNA locations based on the improved K-nearest neighbor (iKNN) classifier. Curr. Bioinform. 15 (6), 11. 10.2174/1574893614666191003142406

[B44] Wu J. Mao X. Cai T. Luo J. Wei L. (2006). KOBAS server: a web-based platform for automated annotation and pathway identification. Nucleic Acids Res. 34 (Web Server issue), W720–W724. 10.1093/nar/gkl167 16845106 PMC1538915

[B45] Wu S. Zhu Z. Fu L. Niu B. Li W. (2011). WebMGA: a customizable web server for fast metagenomic sequence analysis. BMC Genomics 12, 444. 10.1186/1471-2164-12-444 21899761 PMC3180703

[B46] Yang W. Zhu X. J. Huang J. Ding H. Lin H. (2019). A brief survey of machine learning methods in protein sub-Golgi localization. Curr. Bioinform. 14, 7. 10.2174/1574893613666181113131415

[B47] Zhang T. W. R. Jiang Q. Wang Y. (2020). An information gain-based method for evaluating the classification power of features towards identifying. Enhancers Curr. Bioinform. 15 (6), 6. 10.2174/1574893614666191120141032

[B48] Zhou C. E. Smith J. Lam M. Zemla A. Dyer M. D. Slezak T. (2007). MvirDB–a microbial database of protein toxins, virulence factors and antibiotic resistance genes for bio-defence applications. Nucleic Acids Res. 35 (Database issue), D391–D394. 10.1093/nar/gkl791 17090593 PMC1669772

